# Prevalence of Frailty and Its Association with Cognitive Status and Functional Fitness among Ambulating Older Adults Residing in Institutions within West Coast of Peninsular Malaysia

**DOI:** 10.3390/ijerph16234716

**Published:** 2019-11-26

**Authors:** Resshaya Roobini Murukesu, Devinder Kaur Ajit Singh, Ponnusamy Subramaniam, Xee Vern Tan, Ibtisam Arfah Mohamd Izhar, Pavapriya Ponvel, Hanif Farhan Mohd Rasdi

**Affiliations:** 1 Physiotherapy Programme & Centre for Healthy Ageing and Wellness, Faculty of Health Sciences, Universiti Kebangsaan Malaysia, Kuala Lumpur 50300, Malaysia; resshaya@gmail.com (R.R.M.); devinder@ukm.edu.my (D.K.A.S.); xeevern@gmail.com (X.V.T.); ibtisam_izhar@yahoo.com (I.A.M.I.); priyaponvel@hotmail.com (P.P.); 2 Health Psychology Programme & Centre for Healthy Ageing and Wellness, Faculty of Health Sciences, Universiti Kebangsaan Malaysia, Kuala Lumpur 50300, Malaysia; 3 Occupational Therapy Programme & Centre for Rehabilitation & Special Needs, Faculty of Health Sciences, Universiti Kebangsaan Malaysia, Kuala Lumpur 50300, Malaysia; hanif_ot@ukm.edu.my

**Keywords:** aging, frailty, institutionalisation, cognition, functional fitness

## Abstract

Aim: There is limited information about the association between frailty, cognitive status and functional fitness in older adults living in institutions. We aimed to determine the prevalence of frailty and its association with cognitive status and functional fitness among pre-frail and frail Malaysian older adults residing in institutions on the west coast of Peninsular Malaysia. Methods: This study included 302 ambulating Malaysian institutionalised older adults. Frailty was identified using Fried’s frailty criteria. Cognitive status was assessed using the Mini Mental State Examination and Addenbrooke’s Cognitive Examination. Functional fitness was assessed using the Senior Fitness test. The association between frailty groups, cognitive status and functional fitness was analysed using binary logistic regression. Results: Prevalence of frailty, prefrailty and robustness in the older adults was 56.6%, 40.7% and 2.9%, respectively. Frailty was found to be associated with hypertension (OR 2.15, 95% CI: 1.11–4.16, *p* = 0.024), lower cognitive status (Addenbrooke’s Cognitive Examination) (OR 0.98, 95% C.I: 0.96–0.99, *p* = 0.038), and lower dynamic balance and mobility (Timed Up and Go test) (OR 1.09, 95% CI: 1.01–1.16, *p* = 0.024). Conclusion: Frailty is highly prevalent among Malaysian institutionalised older adults. Hypertension, cognitive impairment and lower dynamic balance and mobility were found to be risk factors of frailty. Screening of frailty and its associated factors should be prioritized among institutionalised older adults in view of early prevention and rehabilitation.

## 1. Introduction

Across the globe, the ageing population is rapidly increasing. The number of older adults aged 60 years and above is projected to be 2.1 billion by the year 2050, which is more than double the current population, which was estimated to be 962 million in the year 2017 [1]. The Malaysian national policy defines senior citizens as individuals aged 60 years and above, which is 5 years younger than in developed nations, where older adults are those aged 65 years and above [2]. As of the year 2000, the total population of older adults was estimated to be 6.8%, and this was expected to rise to 10% of the entire nation’s population by the year 2020 [3]. As life expectancy increases, so too does susceptibility to age-related health decline.

Labelled as a ‘frequent consequence’ of ageing, frailty was observed as a common clinical condition, subjecting older adults to accelerated deterioration of health [4,5]. Frailty has been defined as a state of increased vulnerability to stressors (such as acute illness or trauma), resulting in physiological decline, which increases the risk of adverse outcomes [4,6]. The diagnostic criteria of frailty were determined by Fried et al. [7] to be marked by three or more of the following phenotypic criteria: unintentional weight loss in the past year (>5 kg), self-reported exhaustion, weakness (measured by hand grip strength), slow walking speed or low physical activity. 

The prevalence of frailty was found to be increased among those of older age, female gender, institutionalisation, and diagnosed with chronic diseases [8]. A systematic review by Collard et al. [9] reported weighted average of pre-frailty and frailty among community dwelling older adults to be 10.7% and 41.6%, respectively. Institutionalisation of an older adult was observed to have a causal relationship with frailty [10]; however, frailty among institutionalised older adults remains underrepresented. Although the exact aetiology remains unestablished, frailty has been found to manifest due to age-related degeneration of multiple systems in the human body [11]. This debilitating syndrome has been associated with increased risk of falls, injuries, illness, functional disability, hospitalisation and mortality; even more so among those residing in institutions [7]. Ultimately, this leads to limited or even complete functional dependence in carrying out activities of daily living and poor quality of life among older adults with frailty [5,7,12].

Older adults with frailty face difficulties in executing independent functioning which can be understood as functional incompetence. At present, frailty assessments including that of the Cardiovascular Health Study established by Fried et al. [7] focus solely on the physical function components of frailty. However, cognitive impairment has been associated with and suggested to be an inclusion criterion when screening for frailty [13]. In addition, cognitive impairment has been identified to be either an antecedent or an outcome of frailty among older adults [14]. A review by Robertson et al. [15] reported that not only were there higher rates of cognitive impairment among those with frailty as compared to the robust, but also that frailty and dementia could co-occur.

Physical function decline has been labelled a ‘primary pathway’ of the frailty syndrome [16,17]. Although the cause of physical function decline within the frail population cannot be specifically ascribed, progressive muscle damage occurring parallel to biological aging has been associated with physical fragility or disability [17]. The term ‘functional fitness’ has been deemed more fitting when describing the physical function of older adults [18]. Characterised by endurance, strength, agility and flexibility, functional fitness is the basic preserved physical ability to independently and safely execute activities of daily living [19].

An ineluctable relationship between cognitive impairment and physical function performance has been determined, whereby a deterioration in one construct results in the subsequent decline in the other among the general ageing population [20]. However, the prevalence of frailty and its association between the specific domains of cognitive status and functional fitness and frailty among institutionalised older adults remains obscure. Hence, in this study, we aimed to investigate the prevalence of frailty and its association with cognitive status and functional fitness among ambulating older adults residing in institutions on the west coast of Peninsular Malaysia.

## 2. Methods

### 2.1. Study Design and Participants

A cross-sectional study was carried out from the year 2014 until 2016. Older adults residing in institutions across five states representing west coast of Peninsular Malaysia, namely Malacca, Kuala Lumpur, Perak, Kedah and Johor participated in this study via convenience sampling to increase the chance for larger sample to represent west coast of Peninsular Malaysia. This study included Malaysian older adults of both sexes aged 60 years and above, residing in ‘Rumah Seri Kenangan’ institutions. The Rumah Seri Kenangan (RSK) institutions were established throughout Peninsular Malaysia by the Malaysian Department of Social Welfare to provide care for older adults of low socioeconomic background. The institutions are open to Malaysian older adults aged 60 years and above who have no financial support, relatives or heirs, or permanent residence, and who are living without infectious diseases. The RSK is best described as an older adult facility catering to individuals who require care and protection, as well as medical services including therapeutic rehabilitation [21]. Permission to conduct this study at RSK institutions representing the west coast of Peninsular Malaysia was requested from the Department of Social Welfare Malaysia. Once official approval had been obtained, the respective RSKs were contacted and data collection was carried out thereafter. Employees of the RSKs assisted in gathering ambulating residents to participate in the study and organization of the screening.

This study included older adults who were able to ambulate independently (with or without assistive devices). Exclusion criteria were as follows: being wheelchair bound, with amputated limb(s), bedridden, uncorrected hearing and/or visual impairment, major psychiatric illnesses, e.g., psychosis, communication difficulties and refusal to participate. Data collection was carried out by a team of research assistants who underwent a group training session prior to the screening. The research team comprised of final year physiotherapy undergraduates and clinical psychology postgraduates who already have training in assessing physical function and cognitive function respectively. Participants were provided with intermittent breaks and rest between tests. The Department of Social Welfare, Malaysia granted permission for this study (JKKM 100/12/5/2/2014/288). The ethical approval was obtained from the Research Ethics Committee, Universiti Kebangsaan Malaysia (UKM 1.5.3.5/244/NN-148-2014). Participation in this study was voluntary with informed and written consent.

### 2.2. Sociodemographic and Clinical Characteristics

Sociodemographic and clinical data of the participants were obtain using a structured questionnaire that was administered in the form of a face-to-face interview at each institution. Sociodemographic details obtained included age, ethnicity, level of education, body mass index (BMI) and use of walking aid. Clinical history included history of falls within the past 12 months, diabetes mellitus, arthritis, heart disease, hypertension, hypercholesterolemia, asthma, and depression (measured with the Geriatric Depression Scale (GDS) score of more than 5 [22]. Multi-morbidity was categorised as presence of 2 or more chronic diseases [23]. 

### 2.3. Frailty Assessment

Frailty was assessed using the criteria and cut-off points as outlined in the Cardiovascular Health Study [6], consisting of frailty phenotype sub-parameters including unintentional weight loss of more than 4.5 kg in the past 12 months; exhaustion, assessed via self-reported tiredness based on the following two items of the Centre of Epidemiologic Studies Depression scale (CES-D): ‘I felt that everything I did was an effort’ and ‘I could not get going’ [24]; weakness measured by hand grip strength using digital hand dynamometer (Jamar^®^ Plus+, Patternson Medical, IL, USA) to assess upper body muscular strength; slowness measured by the 5 m gait speed test; and physical inactivity assessed using the Physical Activity Scale for Elderly (PASE) [25]. Participants with the presence of one or two criteria were considered pre-frail, and presence of three or more of the criteria were considered frail. Those who did not meet any criteria were considered robust.

### 2.4. Cognitive Status Assessment

Cognitive status was assessed using the Mini Mental State Examination (MMSE) and Addenbrooke’s Cognitive Examination (ACE-III). The MMSE, a measure of global cognition and screening tool for dementia, assessed the sub-domains of orientation, attention, memory, language and visual construction. The total score of the test was calculated out of 30 points, a score of 24 to 30 indicated normal cognition, 19 to 23 indicated mild cognitive impairment, 10 to 18 indicated moderate cognitive impairment, and 9 or less indicated severe cognitive impairment [26]. The MMSE used in this study was validated among older adults in Malaysia [27]. The ACE-III is a measure of functional impairments and screening tool for the risk of dementia comprising of attention, memory, verbal fluency, language and visuospatial ability. Score of the ACE-III was calculated out of 100, with a score of less than 88 indicating risk of dementia [28]. The ACE-III has been reported with good reliability and validity among Malaysian older adults [29].

### 2.5. Functional Fitness Assessment

Functional fitness status was determined using the Senior Fitness test [18]. The two-minute walk test (2MWT) assessed aerobic endurance whereby participants were required to walk as fast as possible for two minutes. Total distances covered in two minutes, pulse rate, blood pressure, oxygen level (SpO2) and Borg scale were recorded. The back-scratch test (BST) assessed upper limb flexibility whereby the participant stood with one hand reaching over shoulder and reached for another palm behind the back as far as possible and the distance between extended middle fingers were recorded. The chair sit and reach test (CSR) assessed lower limb flexibility, whereby participants reached forward in an attempt to touch their toes from a sitting position on the edge of a chair, and the distance between the extended middle finger and the big toe was recorded. The 30 s sit to stand test (30STS) assessed lower limb strength, whereby participants were required to complete as many ‘sit to stands’ with both arms crossed over the chest within 30 s. The timed up and go test (TUG) assessed functional mobility and dynamic balance whereby the time taken to rise from seated position, walk a distance of 3 m at comfortable walking speed with a turn and return back to sitting was recorded. The single leg stance test (SLS) assessed static balance whereby participants were required to stand on the dominant leg as long as possible and timing is recorded once the suspended foot touched the ground. All functional fitness tests were conducted twice, and the mean score was documented for each test except for strength whereby the best score was taken as the result.

### 2.6. Statistical Analysis

Descriptive analysis of the sample characteristics was conducted according to gender and frailty status. Univariate analysis was conducted using a chi square analysis (χ^2^) for categorical variables and one-way Analysis of Variance (ANOVA) for continuous variables. The prevalences of the five Fried’s frailty phenotypes were calculated based on their respective frequencies in each frailty group (pre-frailty and frailty). A binary logistic regression analysis was carried out with pre-frailty and frailty as the dependent variables against sociodemographic factors, clinical characteristics, cognitive status, and functional fitness performance as a measure of association for each variable. The confidence interval was set at 95% and the level of significance was defined as *p* < 0.05. The statistical analysis for this study was carried out using the IBM Statistical Package for Social Science (SPSS) version 22 (IBM, Armonk, NY, USA).

## 3. Results

A total of 302 older adults aged between 60 and 90 years old (mean 68.90 ± 7.24), living in institutions for an average of 4.09 ± 3.85 years, 208 (68.9%) of whom were men participated in this study (Table 1). Ethnically, most participants were Malays (55.6%), followed by Chinese (26.5%) and Indians (17.9%). In terms of marital status, only a quarter of the participants were married, and almost 75% were unmarried, widowed or divorced. Older adults in this study were mostly placed within normal BMI (55.3%); however, frail older adults were observed to fall mainly under the category of underweight (45.2%), whereas pre-frail older adults fell under class I obesity (63.0%). Approximately 12% of the older adults required the use of a walking aid for mobility. More than half of the older adults in these institutions were living with multi-morbidities (61.6%).

When classified into frailty groups according to Fried et al. (2001), eight (2.6%) older adults were ‘robust’, 171 (56.6%) were pre-frail, and 123 (40.7%) were frail in this study (Table 2). Older adults with pre-frailty and frailty had lower education levels (less than 6 years) (*p* < 0.01) and symptoms of depression (*p* < 0.05). Although not found to be statistically significant, frequency of frailty was higher among older women, older age, longer period of living in institutions, and living with multi-morbidities.

**Figure 1:**
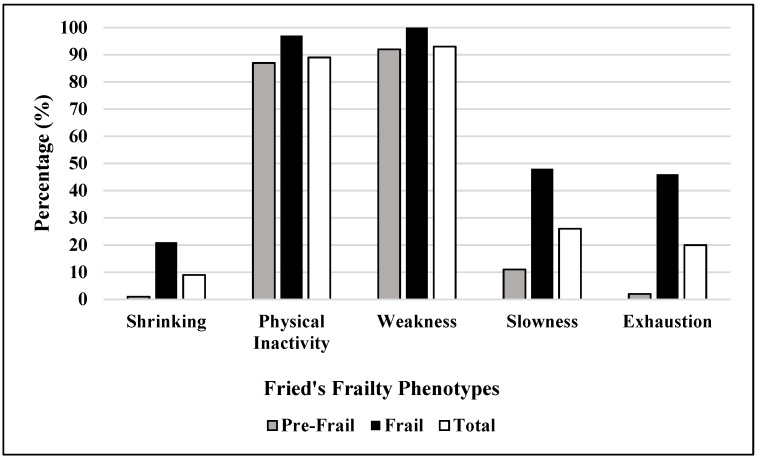
Distribution of frailty phenotype sub-parameters among pre-frail and frail groups.

When the sub-parameters of frailty were considered, weakness was observed to be pre-dominant, with a total percentage of 95.6%, followed by physical inactivity (91.5%), slowness (26.0%), and exhaustion (20.4%), with shrinking being observed to be the least occurring criteria at 9.2% (Figure 1).

In terms of cognitive status, frail older adults had lower MMSE and ACE-III scores as compared to those who were pre-frail or robust, implying lower global cognition (*p* < 0.01) and risk of dementia (*p* < 0.001). Older adults with frailty had generally lower functional fitness with more time taken to complete the TUG test (*p* < 0.001), less area covered with the 2MWT test (*p* < 0.001), and less time sustained in the SLS test (*p* < 0.01) (Table 3).

For the regression analysis, the dependent group consisted of pre-frail and frail older adults. The robust group could not be included in the analysis, as it did not satisfy the minimum requirement of at least 15 cases to 1 variable [30]. The variables included were those observed to be significant in the univariate analysis (Table 4 and Table 5), as well as those that have been found to be significantly associated with frailty (age, gender, ethnicity, body mass index, presence of chronic conditions, depression, multi-morbidity). Malay ethnicity was used as an indicator of the 3 groups, given that they represent the prime population in Malaysia.

Table 6 enumerates the results of the regression. The factors of frailty among institutionalised older adults in this study were enlisted as the following: history of hypertension (*p* < 0.01), ACE-III test (*p* < 0.05) and the TUG test (*p* < 0.05). The presence of a negative B value of the ACE-III test connotes that older adults with lower scores are at a higher risk of frailty. In contrast, the positive B value of the TUG test implies that taking more time for completion increases the odds of frailty.

## 4. Discussion

The prevalence of frailty among older adults residing in institutions on the west coast of Peninsular Malaysia was 40.7%, followed by 56.6% and 2.6% for pre-frail and robust older adults, respectively. The determined prevalence is drastically higher when compared to Malaysian community-dwelling older adults, urban (8.9%) and rural (9.7%), although the prevalence of pre-frailty was observed to be similar, urban (61.7%) and rural (57.9%) [31,32]. However, these findings are in agreement with those reported in a systematic review wherein the prevalence of frailty among institutionalised older adults exhibited an average of 52.3%, followed by pre-frailty at 40.2% [10]. It can be inferred that older adults residing in institutions have lower health outcomes and are not as active as those who are community dwelling; hence, they are more likely to be frail [33].

The regression analysis identified several factors associated with the likelihood of frailty among Malaysian older adults residing in institutions; namely, hypertension, lower ACE-III scores, and longer time taken to complete the TUG test. With respect to clinical factors, the presence of hypertension was identified to increase the risk of frailty. The link between hypertension and frailty has been attributed to less than optimal mobility (defined by walking speed) among older adults with hypertension, which in turn increases the risk of mortality and frailty [34]. A study by Veronese et al. [35] attributed the susceptibility of cardiovascular disease among frail older adults to a higher presence of sub-clinical atherosclerosis. Poor adherence to anti-hypertensive medication among the frail population has also been inferred to be a causative factor [36]. Furthermore, interventions targeted at lowering blood pressure have been shown to reduce the risk of mortality among those living with frailty [37]. Among Malaysians, hypertension is a prevalent chronic disease affecting approximately three-quarters of the population aged 65 and above [38]. Although in this study the presence of multi-morbidities was found to be insignificant, the co-existence of both frailty and hypertension among the older adults asserts the role of chronic disease in increasing the risk of frailty.

Cognitive impairment identified through the ACE-III test was found to be an influential factor in predicting frailty in older adults. In contrast, cognitive status was not identified as a risk factor of frailty among urban Malaysian community-dwelling older adults, but they were observed to be associated among rural older adults [31,32]. The ACE-III was developed with the aim of detecting cognitive impairment, specifically the preliminary stages of dementia and is reported to be a highly sensitive outcome measure in its category [39]. The mean score for older adults in this study implied that the majority of the population had a certain degree of cognitive impairment. The presence of cognitive impairment and dementia has been described as a strong predictor of institutionalisation among older adults [33]. Therefore, it can be deduced that the institutionalisation of older adults in this study could be attributed to a decline in cognitive function. Frailty has been recognized as a risk factor of cognitive dysfunction and risk of dementia [40]. In contrast, the present study suggests that poor cognitive function is a risk factor of frailty, which is in agreement with a study in Singapore, which found an association between history of dementia with frailty [41]. This finding provides further insight into the deep-rooted cyclic relationship between impaired cognition and frailty, whereby the occurrence of one could consequently result in the development of the other [14].

The TUG test is an appropriate measure of functional mobility, as well as dynamic balance among frail older adults [42]. In our study, we identified older adults who took a longer time to complete the TUG test to be at higher risk of frailty, implying that less than optimal functional mobility and dynamic balance results in susceptibility to being frail. This finding is supported by a study conducted among Brazilian older adults, whereby time taken to complete the TUG was significantly longer among frail older adults [43]. Although the parameter of ‘slowness’ measured using the gait speed test is one of the five Fried’s frailty criteria, the TUG test has been identified as a reliable measure of frailty in its own right [44]. The link between impaired functional mobility and dynamic balance with frailty can be explained by the age-related physiological changes that occur in the body with frailty such as sarcopenia resulting in loss of muscle mass and function [45]. A systematic review by Cadore et al. suggested that interventions aimed at addressing frailty should focus on improving balance, gait ability and functional capacity among older adults [46].

The association between functional fitness and frailty is currently an area of interest within geriatric studies in the development of effective prevention and intervention of this syndrome [17]. However, it is noteworthy that older adults with cognitive impairment have been observed to take more time to complete the TUG test among older adults in a local study [47]. Though the aforementioned study was not conducted among frail populations, it can be hypothesized that the less-than-optimal completion time of the TUG test for older adults in the present study may have some connotations with respect to the presence of cognitive impairment. It is vital to consider that the role of cognitive function and functional fitness go hand in hand when addressing a frail older adult. This study finds cognitive impairment and lower functional mobility, a domain of functional fitness to be factors influencing the likelihood of frailty. To ensure optimal functionality of an older adult, both constructs must be evaluated and addressed together rather than separately, as suggested by Kelaiditi et al. based on the recently established ‘cognitive frailty’ syndrome [48].

To the best of our knowledge there has yet to be a study providing insight into the prevalence of frailty, its associated factors, and the cognitive and functional status among the ethnically diverse Malaysian older adults residing in institutions. Identification of influential factors on the frailty syndrome among institutionalised older adults provides a platform for addressing, managing, or potentially reversing the modifiable risk factors with the appropriate interventions. Secondly, the findings of this study are comparable to studies conducted among frail community-dwelling older adults. Addressing the study limitations, a causal relationship between the significant factors observed to be associated with frailty could not be formed due to the cross-sectional study design. Hence, there is a need for more explorative studies among institutionalised older adults. Additionally, this study only considered older adults who were mostly independent in terms of functionality. Thus, our findings may not be able to represent dependent older adults living in institutions. In addition, we would like to address the self-reported nature for the frailty component of ‘exhaustion’ using the CES-D as recommended by Fried et al., as well as the presence of medical conditions, although the outcome measures used in this study have all been deemed valid and reliable. Nonetheless, despite the limitations, the outcomes of this study can be used as a reference in understanding frailty within the institution setting and a platform for future studies to overcome its undesirable consequences. As a recommendation, there is a need to provide routine assessment and intervention for frailty, physical and cognitive status among older adults residing in such institutions. Future research is required to provide evidence for prevention and intervention for frailty, physical and cognitive impairments in older adults residing in these institutions.

## 5. Conclusions

To conclude, we found frailty to be highly prevalent among older adults living in institutions on the west coast of Peninsular Malaysia. The factors influencing frailty were found to be the presence of hypertension, cognitive impairment as well as lower functional mobility and dynamic balance. A comprehensive screening of clinical, cognitive and functional fitness domains is imperative among older adults residing in institutions in order to address the modifiable risk factors of frailty with specifically tailored rehabilitation programs.

## Figures and Tables

**Table 1:** Characteristics of older adults residing in institutions according to gender.

Variable	Total *N* = 302	Male (*n* = 208)	Female (*n* = 94)
**Age** (mean ± s.d.)	68.90 ± 7.24	68.72 ± 6.78	69.30 ± 8.19
**Age Range, *n* (%)**			
60–69	181	124 (59.6)	57 (60.6)
70–79	93	68 (32.7)	25 (26.6)
≥80	28	16 (7.7)	12 (12.8)
**Ethnicity, *n* (%)**			
Malay	168	110 (52.9)	58 (61.7)
Chinese	80	59 (28.4)	21 (22.3)
Indian	54	39 (18.8)	15 (16.0)
**Education, *n* (%)**			
No Education	57	35 (16.8)	22 (23.4)
Primary	151	107 (51.4)	44 (46.8)
Secondary	84	59 (28.4)	25 (26.6)
Tertiary	10	7 (3.4)	3 (3.2)
**Years in Institution** (mean ± s.d.)	3.96 ± 3.77	3.97 ± 4.04	3.96 ± 3.13
**BMI (kg/m^2^)** (mean ± s.d.)	23.28 ± 4.83	22.54 ± 4.32	24.92 ± 5.46
**Multimorbidity, *n* (%)**			
≤1 Chronic Disease	116	74 (63.8)	42 (36.2)
≥2 Chronic Diseases	186	134 (72.0)	52 (28.0)

s.d.: standard deviation, BMI: Body Mass Index.

**Table 2:** Sociodemographic and clinical characteristics according to frailty groups.

Variable	Total *N*	Robust *n* (%)	Pre-Frail *n* (%)	Frail *n* (%)	*p*-Value
**Prevalence**	302	8 (2.6)	171 (56.6)	123 (40.7)	
**Gender**					0.422 ^#^
Male	208	4 (1.9)	121 (58.2)	83 (39.9)	
Female	94	4 (4.3)	50 (53.2)	40 (42.6)	
**Age** (mean ± s.d.)	68.90 ± 7.24	66.00 ± 6.26	68.88 ± 7.45	69.11 ± 7.01	0.502 ^+^
**Age Range**					0.104 ^#^
60–69	181	2 (1.1)	104 (57.5)	75 (41.4)	
70–79	93	6 (6.5)	51 (54.8)	36 (38.7)	
≥80	28	0	16 (57.1)	12 (42.9)	
**Ethnicity**					0.532 ^#^
Malay	168	6 (3.6)	96 (57.1)	66 (39.3)	
Chinese	80	2 (2.5)	47 (58.8)	31 (38.8)	
Indian	54	0	28 (51.9)	26 (48.1)	
**Marital Status**					0.663 ^#^
Married	79	1 (1.3)	46 (58.2)	32 (40.5)	
Unmarried/Widowed/Divorced	223	7 (3.1)	125 (56.1)	91 (40.8)	
**Education Years**					0.011 *^,#^
≤6 Years	208	7 (3.4)	106 (51.0)	95 (45.7)	
>6 Years	94	1 (1.1)	65 (69.1)	28 (29.8)	
**Years in Institution** (mean ± s.d.)	4.09 ± 3.85	3.63 ± 1.77	3.96 ± 3.78	4.3 ± 4.07	0.721 ^+^
**BMI (kg/m^2^)** (mean ± s.d.)	23.28 ± 4.83	21.89 ± 3.24	23.42 ± 4.80	23.18 ± 4.96	0.650 ^+^
**BMI Range (kg/m^2^)**					0.608 ^#^
<18.5 (Underweight)	42	2 (4.8)	21 (50.0)	19 (45.2)	
18.5–24.9 (Healthy Weight)	167	4 (2.4)	98 (58.7)	65 (38.9)	
25.0–24.9 (Overweight)	61	2 (3.3)	32 (52.5)	27 (44.3)	
30.0–34.9 (Class I Obesity)	27	0	17 (63.0)	10 (37.0)	
35.0–39.9 (Class II Obesity)	3	0	3 (100.0)	0	
≥40 (Class III Obesity)	2	0	0	2 (100.0)	
**Walking Aid**					0.160 ^#^
Yes	35	0	16 (45.7)	19 (54.3)	
No	267	8 (3.0)	155 (58.1)	104 (39.0)	
**Medical Conditions**					
History of Falls					0.273 ^#^
Yes	115	1 (0.9)	64 (55.7)	50 (43.5)	
No	187	7 (3.7)	107 (57.2)	73 (39.0)	
Diabetes Mellitus					0.271 ^#^
Yes	70	0	42 (60.0)	28 (40.0)	
No	232	8 (3.4)	129 (55.6)	95 (40.9)	
Arthritis					0.866 ^#^
Yes	8	0	5 (62.5)	3 (37.5)	
No	294	8 (2.7)	166 (56.5)	120 (40.8)	
Heart Disease					
Yes	12	1 (8.3)	5 (41.7)	6 (50.0)	
No	290	7 (2.4)	166 (57.2)	117 (40.3)	
Hypertension					0.278 ^#^
Yes	97	1 (1.0)	60 (61.9)	36 (37.1)	
No	205	7 (3.4)	111 (54.1)	87 (42.4)	
Hypercholesterolaemia					0.714 ^#^
Yes	20	0	11 (55.0)	9 (45.0)	
No	282	8 (2.8)	160 (56.7)	114 (40.4)	
Depression					0.035 *^,#^
Yes	166	2 (1.2)	89 (53.6)	75 (45.2)	
No	136	6 (4.4)	82 (60.3)	48 (35.3)	
**Multimorbidity**					0.672 ^#^
≤1 Chronic Disease	116	2 (1.7)	68 (58.6)	46 (39.7)	
≥2 Chronic Diseases	186	6 (3.2)	103 (55.4)	77 (41.4)	

* *p* < 0.05, ^#^ *p*-value for Chi Square Test, ^+^ *p*-value for Independent Samples T-Test, BMI: Body Mass Index.

**Table 3:** Cognitive and functional fitness performance according to frailty groups.

Variable	Total *N* = 302	Robust (*n* = 8)	Pre-Frail (*n* = 171)	Frail (*n* = 123)	*p*-Value
**Cognitive Status**					
MMSE (mean score ± s.d.)	18.54 ± 6.04	22.25 ± 4.59	20.31 ± 6.72	17.85 ± 6.73	0.002 **^,+^
ACE-III (mean score ± s.d.)	49.56 ± 22.05	66.25 ± 17.56	53.91 ± 22.03	42.47 ± 20.27	<0.001 ***^,+^
**Functional Fitness**					
TUG (s ± s.d.)	12.88 ± 4.82	10.61 ± 3.91	11.90 ± 4.12	14.38 ± 5.36	<0.001 ***^,+^
2MWT (m ± s.d.)	96.79 ± 35.14	107.71 ± 28.77	104.91 ± 31.85	84.79 ± 36.62	<0.001 ***^,+^
30STS (reps ± s.d.)	9.49 ± 3.71	10.50 ± 1.19	9.68 ± 3.83	9.14 ± 3.63	0.385 ^+^
CSR (cm ± s.d.)	−5.76 ± 10.74	−4.01 ± 2.38	−4.82 ± 10.72	−7.18 ± 10.90	0.159 ^+^
BS (cm ± s.d.)	−12.06 ± 17.17	−5.85 ± 14.31	−12.11 ± 15.48	−12.64 ± 16.22	0.585 ^+^
SLS (s ± s.d.)	8.76 ± 7.98	15.66 ± 7.92	9.30 ± 7.98	7.50 ± 7.71	0.008 *^,+^

* *p* < 0.05, ** *p* < 0.01, *** *p* < 0.001, ^+^ *p*-value for Independent Samples *t*-test, MMSE: Mini Mental State Examination, ACE-III: Addenbrooke’s Cognitive Examination, TUG: Timed Up and Go, 2MWT: 2 Minute Walk Test, 30STS: 30 Second Sit to Stand, CSR: Chair Sit and Reach Test. BS: Back Scratch Test, SLS: Single Leg Stand Test, s: seconds, m: metres, cm: centimetres, reps: repetitions, s.d.: standard deviation.

**Table 4:** Sociodemographic and clinical characteristics of pre-frail and frail older adults.

Variable	Total *N*	Pre-Frail *n* (%)	Frail *n* (%)	*p*-Value
**Prevalence**	294	171 (58.2)	123 (41.8)	
**Gender**				0.317 ^#^
Male	204	121 (59.3)	83 (40.7)	
Female	90	54 (57.4)	40 (42.6)	
**Age** (mean ± s.d.)	68.93 ± 7.24	68.81 ± 7.16	69.11 ± 7.01	0.722 ^+^
**Age Range**				0.884 ^#^
60–69	179	104 (58.1)	75 (41.9)	
70–79	87	51 (58.6)	36 (41.4)	
≥80	28	16 (57.1)	12 (42.9)	
**Ethnicity**				0.576 ^#^
Malay	162	96 (59.3)	66 (40.7)	
Chinese	78	47 (60.3)	31 (39.7)	
Indian	54	28 (51.9)	26 (48.1)	
**Marital Status**				0.004 **^,#^
Married	78	46 (59.0)	32 (41.0)	
Unmarried/Widowed/Divorced	216	125 (69.9)	91 (30.1)	
**Education Years**				0.006 **^,#^
≤6 Years	201	106 (52.7)	95 (47.3)	
>6 Years	93	66 (70.2)	28 (29.8)	
**Years in Institution** (mean ± s.d.)	4.10 ± 3.89	3.96 ± 3.76	4.29 ± 4.07	0.469 ^+^
**BMI (kg/m^2^)** (mean ± s.d.)	23.32 ± 4.86	23.42 ± 4.80	23.17 ± 4.96	0.669 ^+^
**BMI Range (kg/m^2^)**				0.304 ^#^
<18.5 (Underweight)	40	21 (52.5)	19 (47.5)	
18.5–24.9 (Healthy Weight)	163	98 (60.1)	65 (39.9)	
25.0–24.9 (Overweight)	59	32 (54.2)	27 (45.8)	
30.0–4.9 (Class I Obesity)	27	17 (63.0)	10 (37.0)	
35.0–39.9 (Class II Obesity)	3	3 (100.0)	0	
≥40 (Class III Obesity)	2	0	2 (100.0)	
**Walking Aid**				0.160 ^#^
Yes	35	16 (45.7)	19 (54.3)	
No	259	155 (58.1)	104 (41.9)	
**Medical Conditions**				
History of Falls				0.260 ^#^
Yes	114	64 (56.1)	50 (43.9)	
No	180	107 (59.4)	73 (40.6)	
Diabetes Mellitus				0.501 ^#^
Yes	70	42 (60.0)	28 (40.0)	
No	224	129 (57.6)	95 (42.4)	
Arthritis				0.866 ^#^
Yes	8	5 (62.5)	3 (37.5)	
No	286	166 (58.0)	120 (42.0)	
Heart Disease				0.285 ^#^
Yes	11	5 (45.5)	6 (54.5)	
No	283	166 (58.7)	117 (41.3)	
Hypertension				0.226 ^#^
Yes	96	60 (62.5)	36 (37.5)	
No	198	111 (56.1)	87 (43.9)	
Hypercholesterolaemia				0.714 ^#^
Yes	20	11 (55.0)	9 (45.0)	
No	274	160 (56.7)	114 (40.4)	
Depression				0.080 ^#^
Yes	164	89 (54.3)	75 (45.7)	
No	130	82 (63.1)	48 (36.9)	
**Multimorbidity**				0.387 ^#^
≤1 Chronic Disease	114	68 (59.6)	46 (40.4)	
≥2 Chronic Diseases	180	103 (57.2)	77 (42.8)	

** *p* < 0.01, ^#^ *p*-value for Chi Square Test, ^+^ *p*-value for Independent Samples *t*-test, BMI: Body Mass Index.

**Table 5:** Cognitive and functional fitness performance of pre-frail and frail older adults.

Variable	Total *N* = 294	Pre-Frail (*n* = 171)	Frail (*n* = 123)	*p*-Value
**Cognitive Status**				
MMSE (mean score ± s.d.)	19.27 ± 6.68	20.31 ± 6.72	17.85 ± 6.73	0.003 **^,+^
ACE-III (mean score ± s.d.)	49.11 ± 22.01	53.91 ± 22.03	42.47 ± 20.27	<0.001 ***^,+^
**Functional Fitness**				
TUG (s ± s.d.)	12.93 ± 4.87	11.90 ± 4.12	14.38 ± 5.36	<0.001 ***^,+^
2MWT (m ± s.d.)	96.49 ± 35.29	104.91 ± 31.85	84.79 ± 36.62	<0.001 ***^,+^
30STS (reps ± s.d.)	9.46 ± 3.76	9.68 ± 3.83	9.14 ± 3.63	0.261 ^+^
SRT (cm ± s.d.)	−5.76 ± 10.74	−4.82 ± 10.72	−7.18 ± 10.90	0.159 ^+^
BST (cm ± s.d.)	−12.33 ± 15.77	−12.11 ± 15.48	−12.64 ± 16.22	0.261 ^+^
SLS (s ± s.d.)	8.57 ± 7.91	9.30 ± 7.98	7.50 ± 7.71	0.008 **^,+^

** *p* < 0.01, *** *p* < 0.001, ^+^ *p*-value for Independent Samples *t*-test, MMSE: Mini Mental State Examination, ACE-III: Addenbrooke’s Cognitive Examination, TUG: Timed Up and Go, 2MWT: 2 Minute Walk Test, 30STS: 30 Second Sit to Stand, CSR: Chair Sit and Reach Test, BS: Back Scratch Test, SLS: Single Leg Stand Test, s: seconds, m: metres, cm: centimetres, reps: repetitions, s.d.: standard deviation.

**Table 6:** Results of the regression analysis among older adults residing in institutions with frailty.

Variable	OR (CI 96%)	β	*p*-Value
**Sociodemography**			
Age (mean ± s.d.)	0.96 (0.93–1.01)	−0.035	0.086
Gender	0.94 (0.49–1.79)	−0.056	0.863
Ethnicity			
Malay	1		
Chinese	0.88 (0.45–1.71)	−0.129	0.704
Indian	0.72 (0.63–1.12)	−0.228	0.359
Marital Status	1.34 (0.72–2.51)	1.342	0.356
Education Years	0.52 (0.268–1.01)	−0.654	0.053
Body Mass Index	0.98 (0.93–1.04)	−0.016	0.570
Clinical Characteristics			
History of Falls	1.25 (0.70–2.24)	0.224	0.452
Diabetes Mellitus	0.79 (0.40–1.74)	−0.242	0.550
Arthritis	0.52 (0.08–3.54)	−0.647	0.507
Heart Disease	2.97 (0.76–11.71)	1.092	0.118
Hypertension	2.15 (1.11–4.16)	0.763	0.024 *
Hypercholesterolaemia	2.06 (0.68–6.23)	0.723	0.200
Depression	1.25 (0.72–2.18)	0.221	0.436
Multimorbidity	1.24 (0.60–2.58)	0.219	0.557
**Cognitive Status**			
ACE-III	0.98 (0.96–0.99)	−0.020	0.038 *
MMSE	0.48 (0.21–1.08)	−0.743	0.076
**Functional Fitness**			
TUG	1.09 (1.01–1.16)	0.081	0.024 *
2MWT	0.99 (0.98–1.01)	−0.009	0.084
SLS	0.97 (0.94–1.01)	−0.026	0.074

* *p* < 0.05, MMSE: Mini Mental State Examination, ACE-III: Addenbrooke’s Cognitive Examination, TUG: Timed Up and Go, 2MWT: 2 Minute Walk Test, SLS: Single Leg Stand Test.

## References

[1] United Nations (2017). World Population Ageing 2017—Highlights.

[2] Official Portal of Public Service Department Malaysia. http://www.jpapencen.gov.my/english/senior_citizen.html.

[3] Mafauzy M. (2000). The problems and challenges of the aging population of Malaysia. Malays. J. Med. Sci..

[4] Clegg A., Young J., Iliffe S., Rikkert M.O., Rockwood K. (2013). Frailty in elderly people. Lancet.

[5] Sourdet S., Rouge-Bugat M.E., Vellas B., Forette F. (2012). Frailty and aging. J. Nutr. Health Aging.

[6] Walston J., Bandeen-Roche K., Buta B., Bergman H., Gill T.M., Morley J.E., Fried L.P., Robinson T.N., Afilalo J., Newman A.B. (2019). Moving frailty toward clinical practice: NIA intramural frailty science symposium summary. J. Am. Geriatr. Soc..

[7] Fried L.P., Tangen C.M., Walston J., Newman A.B., Hirsch C., Gottdiener J., Burke G. (2001). Frailty in older adults: Evidence for a phenotype. J. Gerontol. Ser. A Biol. Sci. Med. Sci..

[8] Choi J., Ahn A., Kim S. (2015). Global Prevalence of physical frailty by Fried’s criteria in community-dwelling elderly with national population-based surveys. J. Am. Med. Dir. Assoc..

[9] Collard R.M., Boter H., Schoevers R.A., Oude-Voshaar R.C. (2012). Prevalence of frailty in community-dwelling older persons: A systematic review. J. Am. Geriatr. Soc..

[10] Kojima G. (2018). Frailty as a Predictor of Nursing Home Placement Among Community-Dwelling Older Adults: A Systematic Review and Meta-analysis. J. Geriatr. Phys. Ther..

[11] Buckinx F., Croisier J.L., Reginster J.Y., Dardenne N., Beaudart C., Slomian J., Leonard S., Bruyère O. (2017). Reliability of muscle strength measures obtained with a hand-held dynamometer in an elderly population. Clin. Physiol. Funct. Imaging.

[12] Gobbens R.J.J., van Assen M.A.L.M. (2017). Associations between multidimensional frailty and quality of life among Dutch older people. Arch. Gerontol. Geriatr..

[13] Jacobs J.M., Cohen A., Ein-Mor E., Maaravi Y., Stessman J. (2011). Frailty, cognitive impairment and mortality among the oldest old. J. Nutr. Health Aging.

[14] Godin J., Armstrong J.J., Rockwood K., Andrew M.K. (2017). Dynamics of Frailty and Cognition after Age 50: Why It Matters that Cognitive Decline is Mostly Seen in Old Age. J. Alzheimers Dis..

[15] Robertson D.A., Savva G.M., Kenny R.A. (2013). Frailty and cognitive impairment-A review of the evidence and causal mechanisms. Ageing Res. Rev..

[16] Peterson M.J., Giuliani C., Morey M.C., Pieper C.F., Evenson K.R., Mercer V., Cohen H.J., Visser M., Brach J.S., Kritchevsky S.B. (2009). Physical activity as a preventative factor for frailty: The health, aging, and body composition study. J. Gerontol. Ser. A Biol. Sci. Med. Sci..

[17] Furtado G., Patricio M., Loureiro M., Teixeira M., Ferreira J.P. (2017). Physical fitness and frailty syndrome in institutionalised older women. Percept. Mot. Ski..

[18] Tuna H.D., Edeer A.O., Malkoc M., Aksakoglu G. (2009). Effect of age and physical activity level on functional fitness in older adults. Eur. Rev. Aging Phys. Act..

[19] Rikli R., Jones J. (1999). Development and validation of a functional fitness test for community-residing older adults. J. Aging Phys. Act..

[20] Ramnath U., Rauch L., Lambert E.V., Kolbe-Alexander T.L. (2018). The relationship between functional status, physical fitness and cognitive performance in physically active older adults: A pilot study. PLoS ONE.

[21] Official Portal Department of Social Welfare. http://www.jkm.gov.my/jkm/index.php?r=portal/left&id=M2k3Q2xST0JJWE9Qa2Z2L253VDl2dz09.

[22] Yesavage J.A., Brink T.L., Rose T.L., Lum O., Huang V., Adey M., Leirer V.O. (1982). Development and validation of a geriatric depression screening scale: A preliminary report. J. Psychiatr. Res..

[23] Wallace E., Salisbury C., Guthrie B., Lewis C., Fahey T., Smith S.M. (2015). Managing patients with multimorbidity in primary care. BMJ.

[24] Radloff L.S. (1977). The CES-D scale: A self-report depression scale for research in the general population. Appl. Psychol. Meas..

[25] Washburn R.A., Smith K.W., Jette A.M., Janney C.A. (1993). The Physical Activity Scale for the Elderly (PASE): Development and evaluation. J. Clin. Epidemiol..

[26] Folstein M., Folstein S. (1975). Mini Mental State: A practical method for grading the cognitive state of patients for the clinician. J. Psychiatr. Res..

[27] Ibrahim N.M., Shohaimi S., Chong H.T., Rahman A.H., Razali R., Esther E. (2009). Validation study of the Mini-Mental State Examination in a Malay-speaking elderly population in Malaysia. Dement. Geriatr. Cogn. Disord..

[28] Hsieh S., Schubert S., Hodges J.R., Hoon C., Mioshi E. (2013). Validation of the Addenbrooke’s Cognitive Examination III in Frontotemporal Dementia and Alzheimer’s Disease. Dement. Geriatr. Cogn. Disord..

[29] Kan K.C., Subramaniam P., Shahrizaila N., Kamaruzzaman S.B., Razali R., Ghazali S.E. (2019). Validation of the Malay Version of Addenbrooke’s Cognitive Examination III in Detecting Mild Cognitive Impairment and Dementia. Dement. Geriatr. Cogn. Disord..

[30] Tabachnick B.G., Fidell L.S. (2013). Using Multivariate Statistics.

[31] Badrasawi M., Shahar S., Singh D.K.A. (2017). Risk Factors of Frailty among Multi-Ethnic Malaysian Older Adults. Int. J. Gerontol..

[32] Ahmad N.S., Hairi N.N., Said M.A., Kamaruzzaman S.B., Choo W.Y., Hairi F., Bulgiba A. (2018). Prevalence, transitions and factors predicting transition between frailty states among rural community-dwelling older adults in Malaysia. PLoS ONE.

[33] Luppa M., Luck T., Weyerer S., König H.H., Brähler E., Riedel-Heller S.G. (2009). Prediction of institutionalization in the elderly. A systematic review. Age Aging.

[34] Odden M.C., Peralta C.A., Haan M.N., Covinsky K.E. (2012). Rethinking the association of high blood pressure with mortality in elderly adults: The impact of frailty. Arch. Intern. Med..

[35] Veronese N., Sigeirsdottir K., Eiriksdottir G., Marques E.A., Chalhoub D., Phillips C.L., Harris T.B. (2017). Frailty and Risk of Cardiovascular Diseases in Older Persons: The Age, Gene/Environment Susceptibility-Reykjavik Study. Rejuv. Res..

[36] Chudiak A., Jankowska-Polańska B., Uchmanowicz I. (2017). Effect of frailty syndrome on treatment compliance in older hypertensive patients. Clin. Interv. Aging.

[37] Whelton P.K., Carey R.M., Aronow W.S., Ovbiagele B., Casey D.E., Smith S.C., Mauri L. (2018). Guideline for the Prevention, Detection, Evaluation, and Management of High Blood Pressure in Adults: A Report of the American College of Cardiology/American Heart Association Task Force on Clinical Practice Guidelines. Hypertension.

[38] Naing C., Yeoh P.N., Wai V.N., Win N.N., Kuan L.P., Aung K. (2016). Hypertension in Malaysia: An analysis of trends from the national surveys 1996 to 2011. Medicine.

[39] Bruno D., Vignaga S.S. (2019). Addenbrooke’s cognitive examination III in the diagnosis of dementia: A critical review. Neuropsychiatr. Dis. Treat..

[40] Borges M.K., Canevelli M., Cesari M., Aprahamian I. (2019). Frailty as a predictor of cognitive disorders: A systematic review and meta-analysis. Front. Med..

[41] Vaingankar J.A., Chong S.A., Abdin E., Picco L., Chua B.Y., Shafie S., Subramaniam M. (2017). Prevalence of frailty and its association with sociodemographic and clinical characteristics, and resource utilization in a population of Singaporean older adults. Geriatr. Gerontol. Int..

[42] Podsiadlo D., Richardson S. (1991). The Timed “Up & Go”: A Test of Basic Functional Mobility for Frail Elderly Persons. J. Am. Geriatr. Soc..

[43] Ansai J.H., Farche A.C.S., Rossi P.G., de Andrade L.P., Nakagawa T.H., Takahashi A.C.M. (2019). Performance of Different Timed Up and Go Subtasks in Frailty Syndrome. J. Geriatr. Phys. Ther..

[44] Savva G.M., Donoghue O.A., Horgan F., O’Regan C., Cronin H., Kenny R.A. (2013). Using timed up-and-go to identify frail members of the older population. J. Gerontol. Ser. A Biol. Sci. Med. Sci..

[45] Vereckei E., Ildiko A.G., Hodinka L. (2018). Sarcopenia, Frailty and Dismobility. Biomed. J. Sci. Tech. Res..

[46] Cadore E.L., Rodríguez-Mañas L., Sinclair A., Izquierdo M. (2013). Effects of different exercise interventions on risk of falls, gait ability, and balance in physically frail older adults: A systematic review. Rejuv. Res..

[47] Ibrahim A., Singh D.K.A., Shahar S. (2017). ‘Timed Up and Go’ test: Age, gender and cognitive impairment stratified normative values of older adults. PLoS ONE.

[48] Kelaiditi E., Cesari M., Canevelli M., van Kan G.A., Ousset P.J., Gillette-Guyonnet S., Ritz P., Duveau F., Soto M.E., Provencher V. (2013). IANA/IAGG. Cognitive frailty: Rational and definition from an (I.A.N.A./I.A.G.G.) international consensus group. J. Nutr. Health Aging.

